# Are all programmed cell death protein 1 inhibitors the same?

**DOI:** 10.3389/fonc.2025.1535030

**Published:** 2025-02-14

**Authors:** Jochen H. Lorch, Stacey Stein, Martin J. Edelman

**Affiliations:** ^1^ Department of Medicine, Hematology/Oncology Division, Robert H. Lurie Comprehensive Cancer Center, Feinberg School of Medicine, Northwestern University, Chicago, IL, United States; ^2^ Department of Medicine, Rutgers Robert Wood Johnson Medical School, New Brunswick, NJ, United States; ^3^ Department of Medical Oncology, Fox Chase Cancer Center, Philadelphia, PA, United States

**Keywords:** PD-1, NSCLC, nasopharyngeal, esophageal, cancer, immunotherapy

## Abstract

Programmed cell death protein 1 (PD-1) inhibitors have revolutionized the treatment of many cancers, seven of which are approved by the US Food and Drug Administration (FDA). No head-to-head phase 3 randomized controlled trials (RCTs) comparing PD-1 inhibitors have been conducted so it remains unknown whether clinically meaningful differences exist between them. Preclinical studies that have directly compared PD-1 inhibitors support a differentiating profile associated with toripalimab compared to pembrolizumab and nivolumab with regard to their PD-1 binding sites, binding orientations, and impact on T cell function. Findings of similar or greater benefit among patients with low/no PD-L1 expression versus high/intermediate PD-L1 expression with toripalimab plus chemotherapy were also observed in advanced nasopharyngeal carcinoma and non-small cell lung cancer for both overall survival and progression-free survival. However, determination of clinically-meaningful differences between PD-1 inhibitors requires sufficiently powered head-to-head RCTs.

## 1 Introduction

Pembrolizumab, nivolumab, cemiplimab, dostarlimab, retifanlimab, toripalimab, and most recently tislelizumab are programmed cell death protein 1 (PD-1) inhibitors approved by the US Food and Drug Administration (FDA). Only one PD-1 inhibitor is approved for the treatment of advanced nasopharyngeal carcinoma (NPC [toripalimab]), while a number of these antibodies are approved for advanced non-small cell lung cancer (NSCLC [pembrolizumab, nivolumab, and cemiplimab]) and esophageal squamous cell carcinoma (ESCC [pembrolizumab, nivolumab, and tislelizumab]). Thus, a total of seven of these agents are available to physicians in the United States. However, no head-to-head phase 3 trials comparing PD-1 inhibitors have been conducted so it remains unknown whether clinically meaningful differences exist between them.

The objective of this manuscript is to analyze available preclinical and clinical data to gain a better understanding as to whether there is preclinical evidence that might distinguish one of the PD-1 inhibitors from the others and if so, to determine whether reported randomized controlled trial (RCT) data might provide evidence of clinically meaningful differences.

## 2 Methodology

All literature searches were performed using PubMed (https://pubmed.ncbi.nlm.nih.gov/) on March 13, 2024. For the preclinical results portion of the manuscript, our goal was to identify studies that could be used to support potential clinical differences across PD-1 inhibitors. Thus, we performed a PubMed search that used the following search string: (pembrolizumab OR nivolumab OR cemiplimab OR dostarlimab OR retifanlimab OR toripalimab OR tislelizumab) AND (epitope OR affinity OR potency).

After evaluating the preclinical data that indicated a possible advantage for toripalimab, we compared published data from phase 3 RCTs that investigated a PD-1 inhibitor plus chemotherapy versus chemotherapy as first-line treatment for advanced NPC, NSCLC, and esophageal squamous carcinoma (ESCC). A literature search was performed for each tumor type separately. For advanced NPC, we used the following search string: (“nasopharyngeal” AND “recurrent”) OR (“nasopharyngeal” AND “metastatic”). For advanced ESCC, we used the following search string: (esophageal cancer OR esophageal carcinoma) AND (pembrolizumab OR nivolumab OR cemiplimab OR dostarlimab OR retifanlimab OR toripalimab OR tislelizumab OR camrelizumab). For advanced NSCLC, we used the following search string: (“non-small cell lung cancer” OR “non-small cell lung carcinoma” OR “NSCLC” OR “carcinoma, non-small-cell lung”) AND (pembrolizumab OR nivolumab OR cemiplimab OR dostarlimab OR retifanlimab OR toripalimab OR tislelizumab OR camrelizumab). Search results for each tumor type were then filtered to only include clinical trials published within the last 10 years with an available abstract.

## 3 Results

### 3.1 Preclinical data comparisons

Crystallization experiments have demonstrated that toripalimab, pembrolizumab, and nivolumab bind different regions of PD-1 (FG, C’D and N-terminal loops, respectively) and that the binding orientation of toripalimab to PD-1 is distinct from that of pembrolizumab) (1). Furthermore, the FG loop of PD-1 which is a critical binding site for PD-L1 and reported “hot spot” for PD-1/programmed death ligand 1 (PD-L1) blockade adopts differing conformations following the binding of these three antibodies (1–3). Whether the differential binding modes reported for toripalimab, pembrolizumab and nivolumab translate into clinically observable differences is unknown.

From a binding kinetics standpoint, target receptor affinity is a critical property affecting the ability of a therapeutic antibody to block ligand binding and subsequent downstream cell signaling (4). As PD-1 inhibitors impart efficacy by blocking activation of PD-1 by its ligands, determining whether the various PD-1 inhibitors display different affinities for PD-1 could provide insight into the existence of potential molecular/mechanistic differences that may translate into clinically observable differences. Results from a recent preclinical comparison of toripalimab to pembrolizumab reported the former exhibited a 12-fold higher binding affinity for human PD-1 (5), findings consistent with results from a previous preclinical comparison of PD-1 inhibitors (6). This study also demonstrated more potent enhancement of human T-cell activation with toripalimab than pembrolizumab across multiple *in vitro* assays as indicated by greater induction of both Th1 (IFN-γ, IL-2, TNF, GM-CSF and IL-18) and myeloid-derived (IL-1α, IL-1β and IL1RA) cytokines, as well as greater activation of human CD8+ T cells (higher IFN-γ secretion) in an assay lacking PD-L1-expressing antigen-presenting or tumor cells. Similar to the differences in PD-1 binding site and orientation associated with these two antibodies discussed in the preceding paragraph, whether the differential target receptor affinities and impact upon *in vitro* T-cell function reported for toripalimab and pembrolizumab translate into clinically observable differences is unknown.

High-resolution crystallography experiments have demonstrated that dostarlimab and pembrolizumab (which have been compared in an RCT (7)) have distinct PD-1 binding sites, and following their binding induce different PD-1 loop conformations (8). In contrast, pharmacokinetic experiments of PD-1 target engagement demonstrated that dostarlimab and pembrolizumab were equipotent as assessed by ex vivo IL-2 stimulation ratios (9). Whether the PD-1 binding site and conformational change differences noted between dostarlimab and pembrolizumab translate into clinically observable differences is unknown, although the similar *in vitro* potency results suggest they would not.

### 3.2 Phase 3 RCT comparisons: advanced NPC

Our literature search for advanced NPC identified a total of 107 publications. Among the total, three phase 3 RCTs that investigated a PD-1 inhibitor plus chemotherapy versus chemotherapy as first-line therapy for recurrent or metastatic NPC were identified: JUPITER-02 (10), RATIONALE-309 (11), and CAPTAIN-1st (12) [**Table 1**]). The chemotherapy regimen in all three trials consisted of cisplatin plus gemcitabine for up to six cycles, followed by maintenance with the PD-1 inhibitor or placebo for up to 2 years. All studies were conducted in Asia. Although not a criterion for study inclusion, most patients had non-keratinizing disease, were positive for Epstein-Barr virus DNA, and had PD-L1 expression ≥1% (CAPTAIN-1st did not contain any information about baseline PD-L1 expression level).

**Table 1:** Phase 3 RCTs investigating a PD-1 inhibitor plus chemotherapy versus chemotherapy for untreated, advanced NPC.

Trial Name	Population	Treatment Arms	OS HR (95% CI)^a^		PFS HR (95% CI)^a^	
			PD-L1 <1%	PD-L1 ≥1%	PD-L1 <1%	PD-L1 ≥1%
JUPITER-02 (10) (NCT03581786) China, Singapore, Taiwan	RM-NPC (N=289)	Toripalimab or placebo + gemcitabine/cisplatin up to 6 cycles; maintenance toripalimab or placebo (toripalimab or placebo for up to a total of 2 years)	0.37 (0.17, 0.80)	0.76 (0.50, 1.15)	0.32 (0.17, 0.62)	0.66 (0.44, 0.99)
RATIONALE-309 (11) (NCT03924986) China, Thailand, Taiwan	RM-NPC (N=263)	Tislelizumab or placebo + gemcitabine/cisplatin up to 6 cycles; maintenance tislelizumab or placebo (tislelizumab or placebo for up to a total of 2 years)	NA	NA	0.47 (0.23, 0.97)	0.47 (0.33, 0.66)
CAPTAIN-1st (12) (NCT03707509) China	RM-NPC (N=263)	Camrelizumab or placebo + gemcitabine/cisplatin up to 6 cycles; maintenance camrelizumab or placebo (camrelizumab or placebo for up to a total of 2 years)	NA	NA	NA	NA

CI, confidence interval; HR, hazard ratio; NA, not available; NPC, nasopharyngeal carcinoma; OS, overall survival; PD-1, programmed cell death protein 1; PD-L1, programmed death ligand 1; PFS, progression-free survival; RCT, randomized controlled trial; RM, recurrent or metastatic.

a PD-1 inhibitor plus chemotherapy arm versus chemotherapy arm.

**Figure 1:**
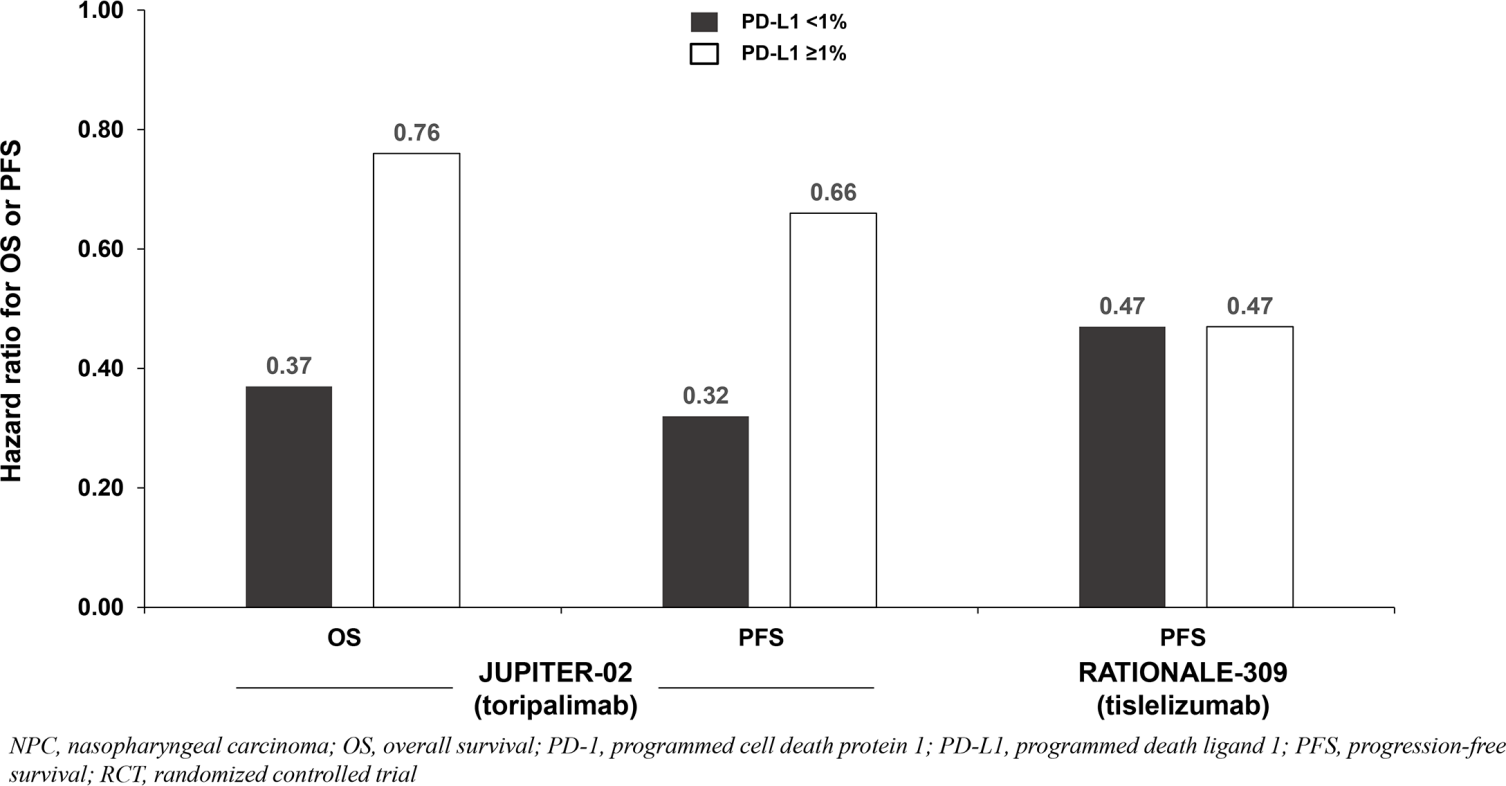
Survival benefit by PD-L1 expression from phase 3 RCTs investigating a PD-1 inhibitor plus chemotherapy vs chemotherapy for untreated, advanced NPC.

All three trials demonstrated significant survival benefit with addition of a PD-1 inhibitor to chemotherapy among the entire patient population (PD-L1-any; **Figure 1**). The hazard ratios (HRs) for both overall survival (OS) and progression-free survival (PFS) were very similar across the three trials (ranges: 0.60 to 0.67 for OS; 0.50 to 0.52 for PFS). Only two of the three studies (JUPITER-02 and RATIONALE-309) reported survival benefit according to PD-L1 expression level. RATIONALE-309 assessed PD-L1 expression on tumor cells only by SP263 immunohistochemistry staining, whereas JUPITER-02 assessed PD-L1 expression on both tumor cells and immune cells using the JS311 assay. These studies demonstrated that adding either toripalimab (JUPITER-02) or tislelizumab (RATIONALE-309) to chemotherapy provided survival benefit independent of PD-L1 expression (**Table 1**; **Figure 1**). In RATIONALE-309, PFS benefit was highly consistent across the entire trial population (PD-L1-any) and PD-L1-positive/-negative subgroups (≥1% versus <1%) with HRs of 0.50, 0.47, and 0.47, respectively (OS benefit by PD-L1 expression level was not reported). In contrast to RATIONALE-309, OS and PFS benefit in JUPITER-02 were greater in the PD-L1-negative (<1%) subgroup versus both the PD-L1-positive (≥1%) subgroup and the entire trial population (PD-L1-any); the reason(s) for these seemingly counterintuitive findings are unknown although it may be that the efficacy of PD-1 inhibitors combined with chemotherapy in recurrent or metastatic NPC is relatively independent of PD-L1 expression. Collectively, the data demonstrate that adding either toripalimab, tislelizumab or camrelizumab to chemotherapy provides survival benefit in untreated recurrent or metastatic NPC, and the survival benefit of adding toripalimab or tislelizumab to chemotherapy appears to be independent of baseline PD-L1 expression level. Whether these findings apply to other PD-1 inhibitors remains unknown.

### 3.3 Phase 3 RCT comparisons: advanced ESCC

Our literature search for advanced ESCC identified a total of 107 publications. Among the total, only five phase 3 RCTs that investigated a PD-1 inhibitor plus chemotherapy versus chemotherapy as first-line therapy for advanced ESCC were identified: JUPITER-06 (13), RATIONALE-306 (14), ESCORT-1st (15), CheckMate 648 (16), and KEYNOTE-590 (17) (**Table 2**). The chemotherapy regimen varied across trials (all included a platinum, but the partner varied [fluoropyrimidine or paclitaxel]). The maintenance regimen varied across trials as well (most trials only continued the PD-1 inhibitor while RATIONALE-306 also continued chemotherapy). Three studies were global versus two conducted exclusively in China. There were also cross-trial differences in baseline PD-L1 expression levels, assays used to determine PD-L1 expression, and the cell populations assessed for PD-L1 expression (tumor cells only versus combined tumor and immune cells). Three trials (RATIONALE-306, ESCORT-1st, CheckMate 648) reported that ~50% of the entire population were PD-L1-positive (≥1%) as assessed on tumor cells. JUPITER-06 reported the highest proportion of the population that were PD-L1-positive at baseline (78%) although PD-L1 was assessed on both tumor and immune cells. KEYNOTE-590 did not report the proportion of the ESCC population that was PD-L1-positive (≥1%) at baseline, but 52% had a combined positive score ≥10% as assessed on both tumor and immune cells.

**Table 2:** Phase 3 RCTs investigating a PD-1 inhibitor plus chemotherapy versus chemotherapy for untreated, advanced ESCC.

Trial Name	Population	Treatment Arms	OS HR (95% CI)^a^				PFS HR (95% CI)^a^			
			PD-L1 <1%	PD-L1 ≥1%	PD-L1 <10%	PD-L1 ≥10%	PD-L1 <1%	PD-L1 ≥1%	PD-L1 <10%	PD-L1 ≥10%
JUPITER-06 (13) (NCT03829969) China	Locally advanced, recurrent or metastatic ESCC (N=514)	Toripalimab or placebo + cisplatin/paclitaxel up to 6 cycles; maintenance toripalimab or placebo (toripalimab or placebo for up to a total of 2 years)	**0.61** (0.30, 1.25)	**0.61** (0.44, 0.87)	**0.61** (0.40, 0.93)	**0.64** (0.40, 1.03)	**0.66** (0.37, 1.19)	**0.58** (0.44, 0.75)	**0.56** (0.41, 0.78)	**0.65** (0.45, 0.92)
RATIONALE-306 (14) (NCT03783442) Global	Locally advanced, recurrent or metastatic ESCC (N=649)	Tislelizumab or placebo + (cisplatin or oxaliplatin)/ (5-FU or capecitabine or paclitaxel) for up to a total of 2 years	**0.79** (0.57, 1.09)	**0.65** (0.49, 0.87)	**0.82** (0.62, 1.08)	**0.57** (0.41, 0.80)	NA	NA	NA	NA
ESCORT-1st (15) (NCT03691090) China	Locally advanced, recurrent or metastatic ESCC (N=596)	Camrelizumab or placebo + cisplatin/paclitaxel up to 6 cycles; maintenance camrelizumab or placebo	**0.79** (0.57, 1.11)	**0.59** (0.43, 0.80)	**0.78** (0.59, 1.02)	**0.52** (0.35, 0.79)	**0.62** (0.46, 0.83)	**0.51** (0.39, 0.67)	**0.59** (0.46, 0.75)	**0.51** (0.36, 0.72)
CheckMate 648 (16) (NCT03143153) Global	Locally advanced, recurrent or metastatic ESCC (N=970 [645^b^])	Nivolumab or placebo + cisplatin/fluorouracil for up to a total of 2 years	**0.98** (0.76, 1.28)	**0.55** (0.42, 0.72)	**0.79** (0.63, 0.99)	**0.62** (0.44, 0.87)	**0.95** (0.73, 1.24)	**0.65** (0.46, 0.92)	NA	NA
KEYNOTE-590 (17) (NCT03189719) Global	Locally advanced or metastatic ESCC (N=749 [548^c^])	Pembrolizumab or placebo + cisplatin/5-FU up to 6 cycles; maintenance pembrolizumab or placebo (pembrolizumab or placebo for up to a total of 2 years)	NA	NA	**0.99** (0.74, 1.32)	**0.57** (0.43, 0.75)	NA	NA	**0.83** (0.64, 1.10)	**0.53** (0.40, 0.69)
FDA Pooled Analysis of RATIONALE-306, CheckMate 648 and KEYNOTE-590 (18)	Locally advanced, recurrent or metastatic ESCC (N=1,825)	PD-1 inhibitor (tislelizumab, nivolumab or pembrolizumab) or placebo + chemotherapy for up to a total of 2 years	**1.1** (0.76, 1.58)	**0.68** (0.60, 0.77)	**0.82** (0.70, 0.96)	**0.61** (0.52, 0.73)	NA	NA	NA	NA

5-FU, 5-fluorouracil; CI, confidence interval; ESCC, esophageal squamous cell carcinoma; HR, hazard ratio; NA, not available; OS, overall survival; PD-1, programmed cell death protein 1; PD-L1, programmed death ligand 1; PFS, progression-free survival; RCT, randomized controlled trial.

a PD-1 inhibitor plus chemotherapy arm versus chemotherapy arm.

b N value excluding the nivolumab plus ipilimumab arm.

c Value of ESCC population excluding adenocarcinoma.

**Figure 2:**
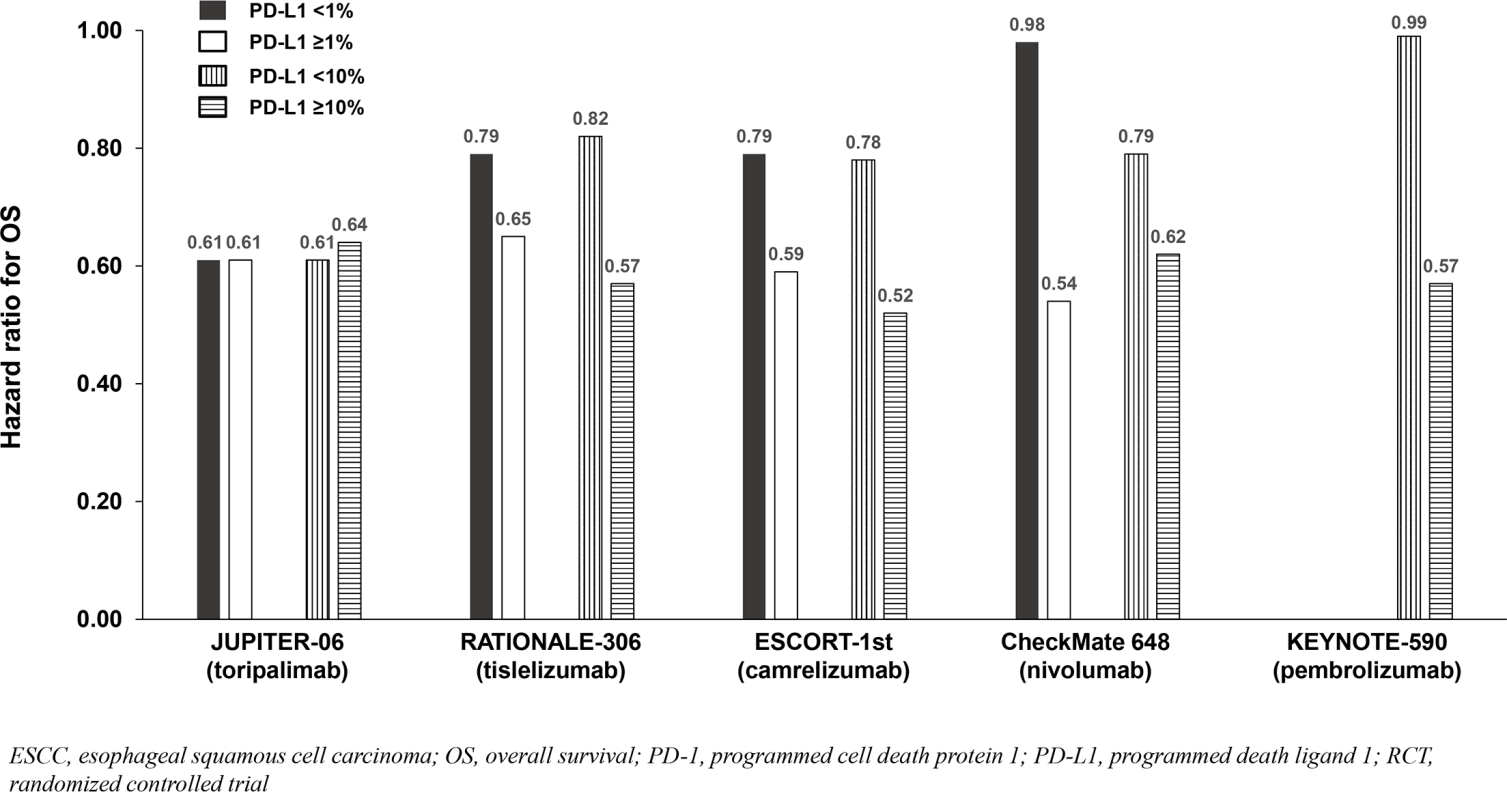
OS benefit by PD-L1 expression from phase 3 RCTs investigating a PD-1 inhibitor plus chemotherapy vs chemotherapy for untreated, advanced ESCC.

**Figure 3:**
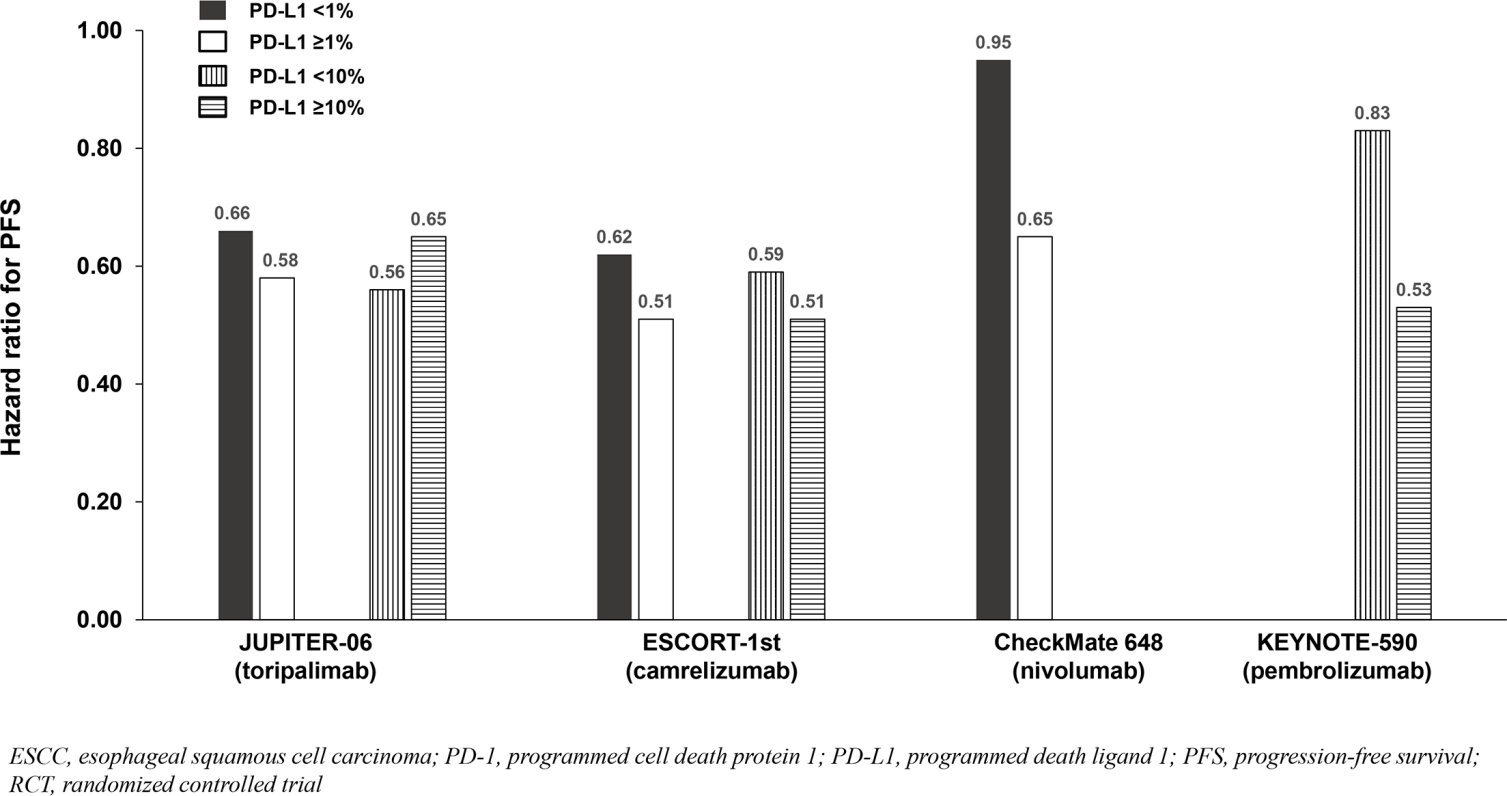
PFS benefit by PD-L1 expression from phase 3 RCTs investigating a PD-1 inhibitor plus chemotherapy vs chemotherapy for untreated, advanced ESCC.

All trials demonstrated significant survival benefit with addition of a PD-1 inhibitor to chemotherapy among the entire population (PD-L1-any; **Figure 2**). The HRs for OS were similar across trials (0.58 to 0.74). Similarly, the HRs for PFS were similar across trials (0.56 to 0.65) except for CheckMate 648 (0.81). Among the five identified studies in advanced ESCC, four reported survival benefit that correlated with PD-L1 expression level whereby PD-L1-positive (≥1%) patients achieved greater benefit than PD-L1-negative (<1%) patients, and patients with intermediate/high PD-L1 (≥10%) benefited more than those with low/no PD-L1 expression (<10%). Among these four studies, RATIONALE-306 and ESCORT-1st reported less OS benefit among the subgroups with PD-L1 <1% versus ≥1% and <10% versus ≥10%, while CheckMate648 and KEYNOTE-590 reported little-to-no benefit among the subgroups with PD-L1 <1% and <10%, respectively (**Table 2**; **Figure 2**). These findings are consistent with a pooled analysis of patient-level OS data from three of these trials (RATIONALE-306, CheckMate648, and KEYNOTE-590) conducted by the FDA and presented at the September 26, 2024 Oncologic Drugs Advisory Committee meeting which demonstrated no clear benefit among patients with PD-L1 <1 (18). JUPITER-06 which investigated toripalimab plus chemotherapy was the only trial that reported an OS HR and 95% confidence interval (CI) that were below 1.0 among patients with PD-L1 <10, and perhaps just as notably, was the only trial that reported an OS HR that was nearly identical across subgroups with PD-L1 <1, ≥1, <10 and ≥10 indicating that benefit was independent of PD-L1 expression level. Among the three studies that reported PFS results among the population with PD-L1 <1%, the HRs were 0.62 in ESCORT-1st, 0.66 in JUPITER-06 and 0.95 in CheckMate 648 (**Table 2**; **Figure 3**). JUPITER-06 had the most similar HRs for PFS among the subgroups with PD-L1 <1% versus ≥1% (0.66 versus 0.58) and also reported similar PFS HRs among the subgroups with PD-L1 <10% versus ≥10% (0.56 versus 0.65). PFS HRs were also similar across PD-L1 subgroups in ESCORT-1st (<1%, ≥1%, <10% and ≥10%: 0.62, 0.51, 0.59 and 0.51). In contrast, CheckMate 648 only reported PFS benefit among the subgroup with PD-L1 ≥1% (no benefit among the subgroup with PD-L1 <1% [HRs: 0.65 and 0.95, respectively]) with similar results demonstrated in KEYNOTE-590 using PD-L1 cutoffs of ≥10% and <10% (HRs: 0.53 and 0.83, respectively). The results from JUPITER-06 demonstrating survival benefit with toripalimab plus chemotherapy independent of PD-L1 expression were similar to the results of JUPITER-02 in advanced NPC.

### 3.4 Phase 3 RCT comparisons: advanced NSCLC

Our literature search for advanced NSCLC identified a total of 316 publications. Among the total, six phase 3 RCTs that investigated a PD-1 inhibitor plus chemotherapy versus chemotherapy as first-line treatment for advanced nonsquamous (NSQ) NSCLC were identified: CameL (19), CheckMate 227 Part 2 (20), EMPOWER Lung-3 (21), KEYNOTE-189 (22), CHOICE-01 (23), and RATIONALE 304 (24) (**Table 3**).

**Table 3:** Phase 3 RCTs investigating a PD-1 inhibitor plus chemotherapy versus chemotherapy for untreated, advanced nonsquamous NSCLC.

Trial Name	Population	Treatment Arms	OS HR (95% CI)^a^				PFS HR (95% CI)^a^			
			PD-L1 <1%	PD-L1 ≥1%	PD-L1 1-49%	PD-L1 ≥50%	PD-L1 <1%	PD-L1 ≥1%	PD-L1 1-49%	PD-L1 ≥50%
CameL (19) (NCT03134872) China	Locally advanced or metastatic NSQ (N=412)	Camrelizumab/carboplatin/ pemetrexed vs carboplatin/pemetrexed up to 6 cycles; maintenance camrelizumab/pemetrexed or pemetrexed up to 2 years in total	**0.84** (0.55, 1.27)	**0.70** (0.51, 0.97)	**0.76** (0.53, 1.08)	**0.68** (0.28, 1.63)	**0.75** (0.50, 1.13)	**0.52** (0.39, 0.69)	**0.59** (0.42, 0.81)	**0.42** (0.19, 0.87)
CheckMate 227 Part 2 (20) (NCT02477826) Global	Metastatic NSQ (N=543)	Nivolumab/pemetrexed/ (cisplatin or carboplatin) vs pemetrexed/(cisplatin or carboplatin) up to 4 cycles; maintenance nivolumab/(pemetrexed optional) or pemetrexed (optional) up to 35 cycles in total	**0.91** (0.66, 1.25)	**0.76** (0.55, 1.05)	NA	**0.56** (0.34, 0.92)	NA	NA	NA	NA
EMPOWER Lung-3 (21) (NCT03409614) Global	Locally advanced or metastatic NSQ (N=266)	Cemiplimab or placebo + pemetrexed/(cisplatin or carboplatin) up to 4 cycles; maintenance cemiplimab/pemetrexed or pemetrexed up to 36 cycles in total	**1.26** (0.74, 2.12)	NA	**0.48** (0.28, 0.82)	**0.42** (0.23, 0.76)	**0.79** (0.49, 1.30)	NA	**0.42** (0.26, 0.69)	**0.46** (0.27, 0.80)
KEYNOTE-189 (22) (NCT02578680) Global	Metastatic NSQ (N=616)	Pembrolizumab or placebo + pemetrexed/(cisplatin or carboplatin) up to 4 cycles; maintenance pembrolizumab/pemetrexed or pemetrexed up to 35 cycles in total	**0.55** (0.39, 0.76)	NA	**0.65** (0.46, 0.90)	**0.68** (0.49, 0.96)	**0.67** (0.49, 0.92)	NA	**0.57** (0.41, 0.80)	**0.35** (0.25, 0.49)
CHOICE-01 (23) (NCT03856411) China	Locally advanced or metastatic NSQ (N=245)	Toripalimab or placebo + pemetrexed/(cisplatin or carboplatin) up to 6 cycles; maintenance toripalimab/pemetrexed or pemetrexed up to 2 years in total	NA	NA	NA	NA	NA	NA	NA	NA
RATIONALE 304 (24) (NCT03663205) China	Locally advanced or metastatic NSQ (N=334)	Tislelizumab/pemetrexed/ (cisplatin or carboplatin) up to 6 cycles; maintenance tislelizumab/pemetrexed or pemetrexed	NA	NA	NA	NA	**0.76** (0.47, 1.22)	**0.55** (0.35, 0.87)	**1.06** (0.51, 2.21)	**0.31** (0.17, 0.57)

CI, confidence interval; HR, hazard ratio; NA, not available; NSQ, nonsquamous; OS, overall survival; PD-1, programmed cell death protein 1; PD-L1, programmed death ligand 1; PFS, progression-free survival; RCT, randomized controlled trial

a PD-1 inhibitor plus chemotherapy arm versus chemotherapy arm.

In all six studies, the induction chemotherapy regimen included a platinum plus pemetrexed, but cycle numbers varied from four to six and the maintenance regimen included the PD-1 inhibitor plus pemetrexed in all except maintenance pemetrexed was optional in CheckMate 227 Part 2. Three studies were global versus three conducted exclusively in China. Baseline PD-L1 expression levels were similar, although there were cross-trial differences in the assays used to determine PD-L1 expression.

**Figure 4:**
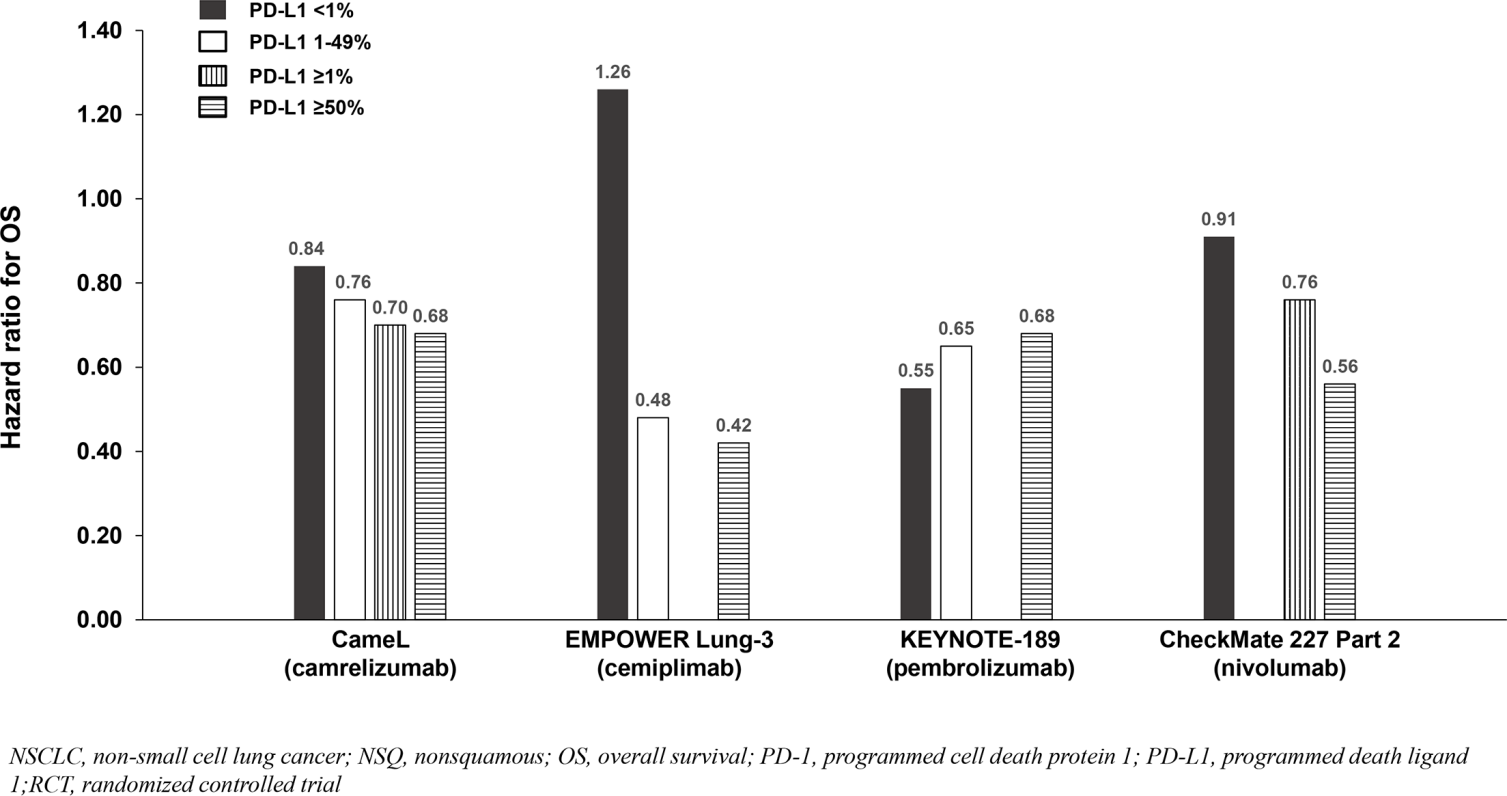
OS benefit by PD-L1 expression from phase 3 RCTs investigating a PD-1 inhibitor plus chemotherapy vs chemotherapy for untreated, advanced NSQ NSCLC.

**Figure 5:**
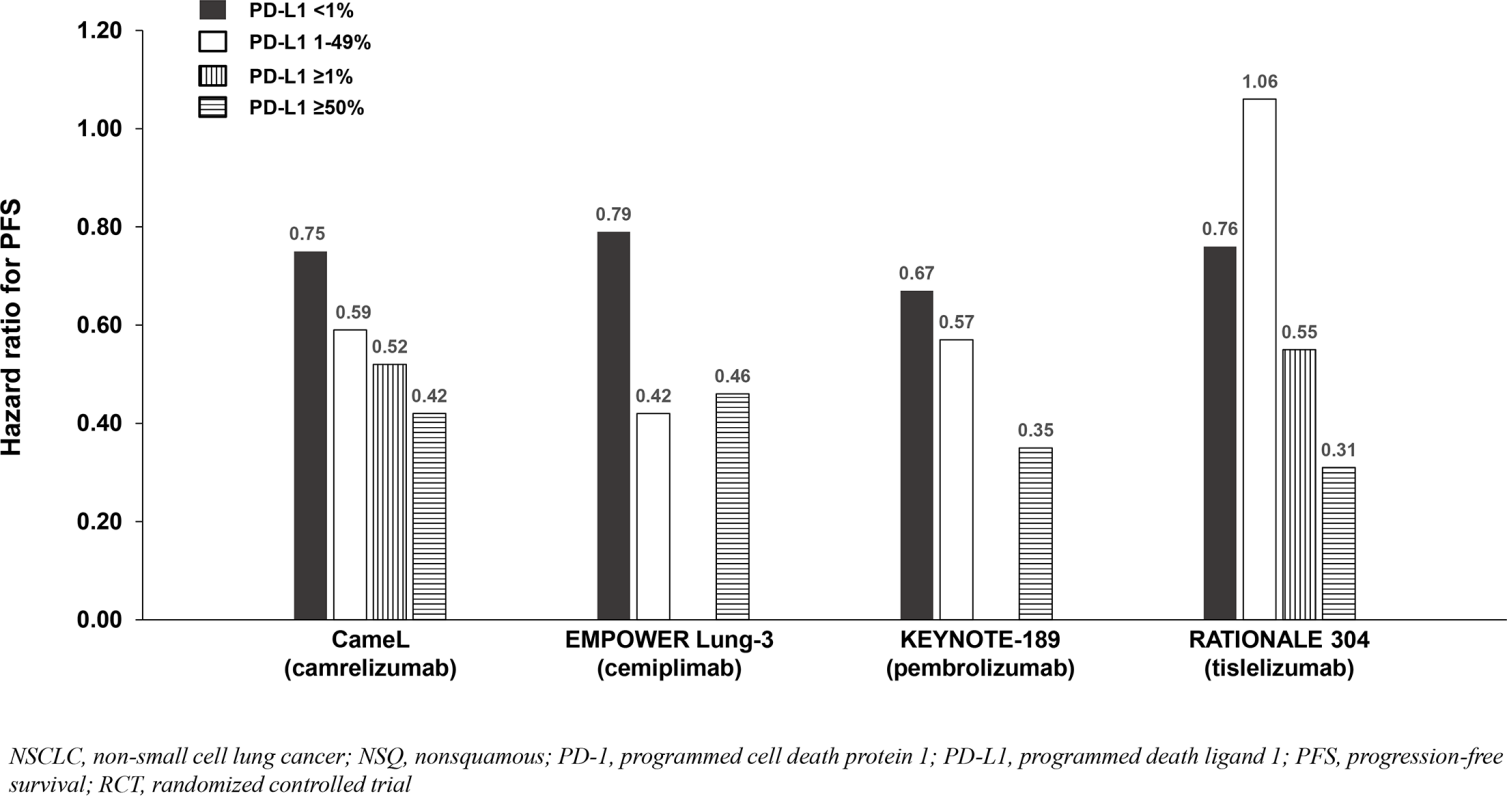
PFS benefit by PD-L1 expression from phase 3 RCTs investigating a PD-1 inhibitor plus chemotherapy vs chemotherapy for untreated, advanced NSQ NSCLC.

Four of six NSQ NSCLC trials demonstrated significant OS benefit with addition of a PD-1 inhibitor to chemotherapy among the entire population (not significant in CheckMate 227 Part 2 and not reported in RATIONALE 304), and PFS benefit reported in all six trials. Across the four trials demonstrating OS benefit, the HRs for OS were similar (0.48 to 0.72) and correlated with PD-L1 expression such that the PD-L1 <1% subgroups achieved less/no benefit in comparison to chemotherapy except in KEYNOTE-189 which demonstrated consistent benefit across the <1%, 1-49% and ≥50% subgroups (**Table 3** **;** **Figure 4**). Due to notable inter-trial differences in the percentage of patients in the control arms of the various trials who received immunotherapy following progression (i.e., crossover to the active treatment or initiation of a different immunotherapy) and the confounding impact it could have on OS results, we also analyzed PFS benefit across the PD-L1 expression level subgroups to identify potential differences between PD-1 inhibitors because this endpoint is not affected by receipt of subsequent immunotherapy. Similar to the OS results across PD-L1 expression subgroups, HRs for PFS correlated with PD-L1 expression such that the PD-L1 <1% subgroups achieved less benefit across trials (**Table 3** **;** **Figure 5** **).**

**Figure 6:**
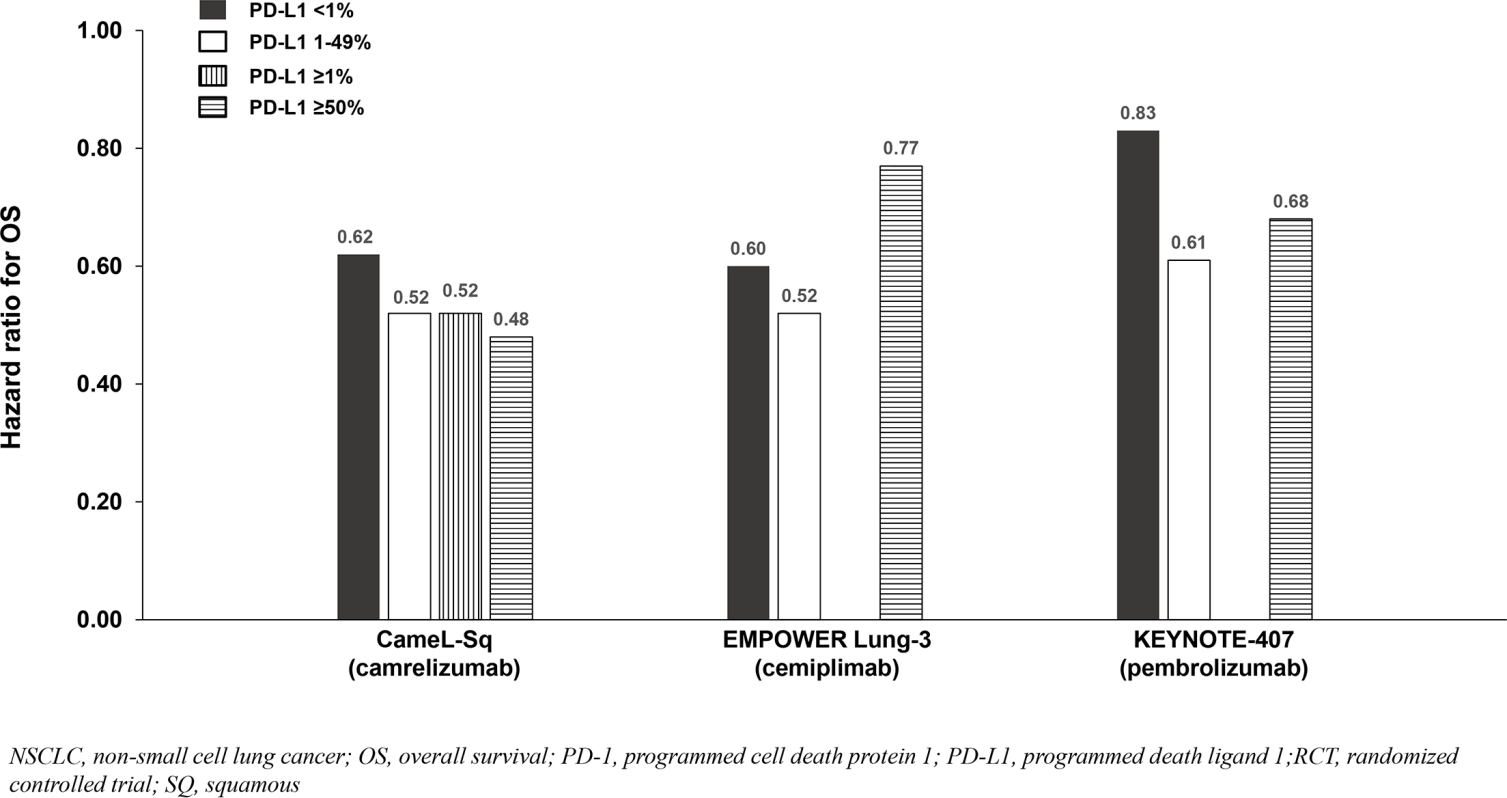
OS benefit by PD-L1 expression from phase 3 RCTs investigating a PD-1 inhibitor plus chemotherapy vs chemotherapy for untreated, advanced SQ NSCLC.

**Figure 7:**
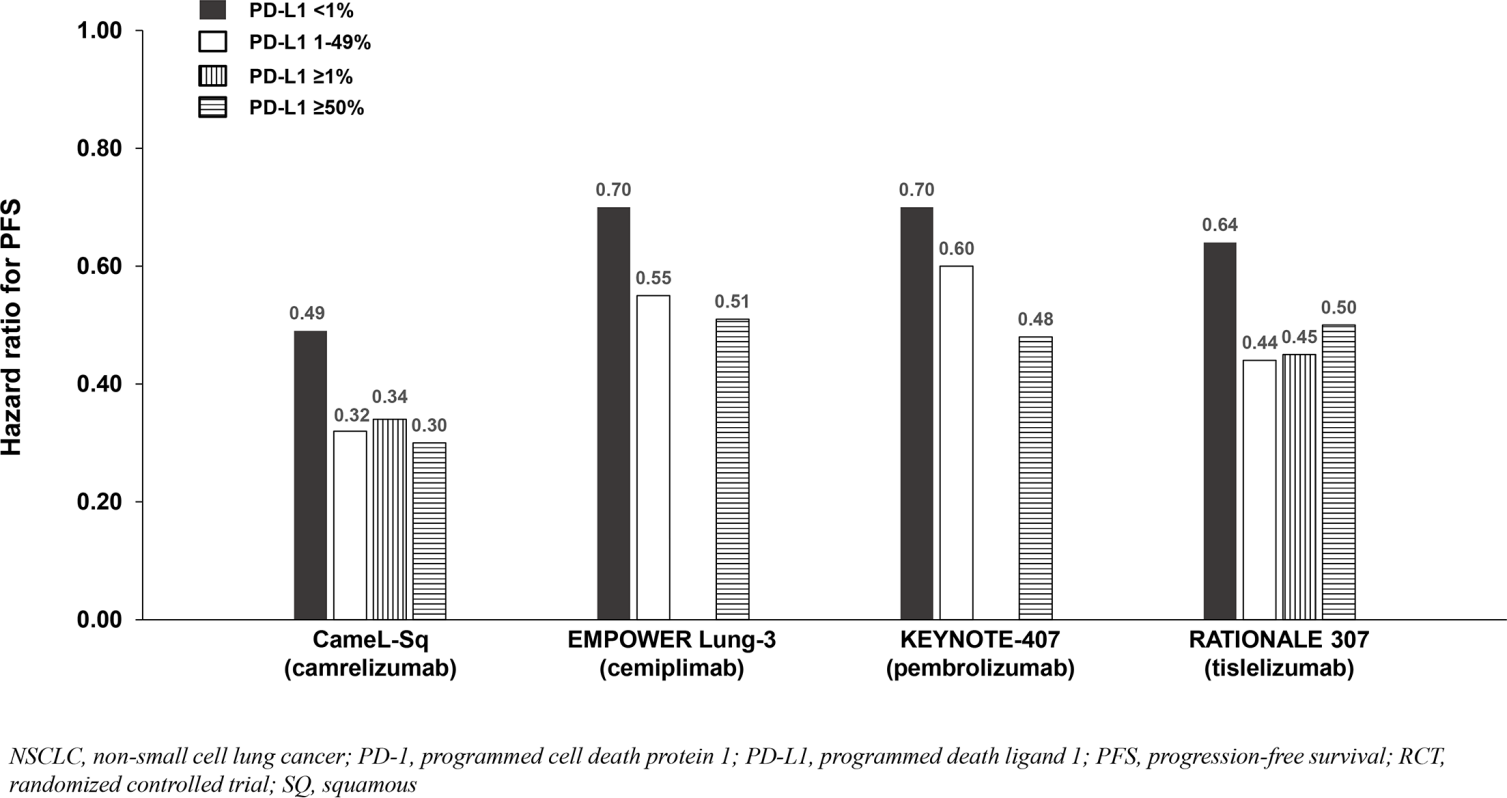
PFS benefit by PD-L1 expression from phase 3 RCTs investigating a PD-1 inhibitor plus chemotherapy vs chemotherapy for untreated, advanced SQ NSCLC.

Six phase 3 RCTs that investigated a PD-1 inhibitor plus chemotherapy versus chemotherapy as first-line therapy for advanced squamous (SQ) NSCLC were identified: CameL-Sq (25), CheckMate 227 Part 2 (20), EMPOWER Lung-3 (21), KEYNOTE-407 (26), CHOICE-01 (23), and RATIONALE 307 (27) (**Table 4**; **Figure 6**). All trials reported an OS benefit among the entire SQ NSCLC population except for CHOICE-01 which had the highest control group crossover rate to immunotherapy upon progression (71%) potentially impacting OS results. All six studies, including CHOICE-01, reported substantial PFS benefit with a PD-1 inhibitor added to chemotherapy (HRs: 0.37 to 0.62; **Figure 7**).

**Table 4:** Phase 3 RCTs investigating a PD-1 inhibitor plus chemotherapy versus chemotherapy for untreated, advanced squamous NSCLC.

Trial Name	Population	Treatment Arms	OS HR (95% CI)^a^				PFS HR (95% CI)^a^			
			PD-L1 <1%	PD-L1 ≥1%	PD-L1 1-49%	PD-L1 ≥50%	PD-L1 <1%	PD-L1 ≥1%	PD-L1 1-49%	PD-L1 ≥50%
CameL-Sq (25) (NCT03668496) China	Locally advanced or metastatic SQ (N=390)	Camrelizumab + carboplatin/paclitaxel vs carboplatin/paclitaxel up to 6 cycles; maintenance camrelizumab up to 2 years in total	**0.62** (0.41, 0.94)	**0.52** (0.31 0.86)	**0.52** (0.27, 1.00)	**0.48** (0.21, 1.12)	**0.49** (0.35, 0.68)	**0.34** (0.24, 0.49)	**0.32** (0.20, 0.51)	**0.30** (0.17, 0.55)
CheckMate 227 Part 2 (20) (NCT02477826) Global	Metastatic SQ (N=212)	Nivolumab/paclitaxel/carboplatin vs paclitaxel/carboplatin up to 4 cycles; maintenance nivolumab up to 35 cycles in total	NA	NA	NA	NA	NA	NA	NA	NA
EMPOWER Lung-3 (21) (NCT03409614) Global	Locally advanced or metastatic SQ (N=200)	Cemiplimab or placebo + paclitaxel/(cisplatin or carboplatin) up to 4 cycles; maintenance cemiplimab or placebo up to 36 cycles in total	**0.60** (0.30, 1.20)	NA	**0.52** (0.29, 0.92)	**0.77** (0.40, 1.45)	**0.70** (0.37, 1.32)	NA	**0.55** (0.33, 0.90)	**0.51** (0.28, 0.92)
KEYNOTE-407 (26) (NCT02775435) Global	Metastatic SQ (N=559)	Pembrolizumab or placebo + (paclitaxel or nab-paclitaxel)/carboplatin up to 4 cycles; maintenance pembrolizumab up to 35 cycles in total	**0.83** (0.61, 1.13)	NA	**0.61** (0.45, 0.83)	**0.68** (0.47, 0.97)	**0.70** (0.52, 0.95)	NA	**0.60** (0.45, 0.81)	**0.48** (0.33, 0.69)
CHOICE-01 (23) (NCT03856411) China	Locally advanced or metastatic SQ (N=220)	Toripalimab or placebo + nab-paclitaxel/carboplatin up to 6 cycles; maintenance toripalimab or placebo up to 2 years in total	NA	NA	NA	NA	NA	NA	NA	NA
RATIONALE 307 (27) (NCT03594747) China	Locally advanced or metastatic SQ (N=360 [241^b^)	Tislelizumab/(nab-paclitaxel or paclitaxel)/carboplatin vs paclitaxel/carboplatin up to 6 cycles; maintenance tislelizumab	NA	NA	NA	NA	**0.64** (0.37, 1.10)^b^	**0.45** (0.29, 0.70)^b^	**0.44** (0.22, 0.87)^b^	**0.50** (0.28, 0.89)^b^

CI, confidence interval; HR, hazard ratio; NA, not available; SQ, squamous; OS, overall survival; PD-1, programmed cell death protein 1; PD-L1, programmed death ligand 1; PFS, progression-free survival; RCT, randomized controlled trial.

a PD-1 inhibitor plus chemotherapy arm versus chemotherapy arm.

b Tislelizumab/paclitaxel versus paclitaxel.

Findings from the studies in advanced SQ NSCLC were similar to those in advanced NSQ NSCLC (and advanced ESCC) whereby most reported that survival benefit correlated with PD-L1 expression level. Among patients with PD-L1 <1%, a relatively smaller OS benefit was observed in two of three SQ NSCLC studies reporting PD-L1 subgroup OS data (**Table 4**; **Figure 6**), and a relatively smaller PFS benefit was observed in all four studies reporting PD-L1 subgroup data (**Table 4**; **Figure 7**).

Among the studies in advanced NSCLC that we identified, only pembrolizumab plus chemotherapy (KEYNOTE-189 [NSQ NSCLC]) reported an OS HR and 95% CI that were below 1.0 among patients with PD-L1 <1% and similar to that observed in the subgroups with PD-L1 1 - 49% and ≥50% (**Tables 3**, **4**, **Figures 4**, **5**, **6**, **7**). Interestingly, these findings were not corroborated by the PFS subgroup data from KEYNOTE-189 which displayed a correlation between benefit and PD-L1 expression level, or by the results of KEYNOTE-407 with pembrolizumab plus chemotherapy in SQ NSCLC which also reported a dose-relationship between PD-L1 expression level and survival benefit (for both OS and PFS).

Unfortunately, we could not assess whether toripalimab plus chemotherapy provided survival benefit independent of PD-L1 expression level in advanced SQ or NSQ NSCLC because the CHOICE-01 publication did not report survival results by PD-L1 expression within each histology (23). However, results for both histologies combined revealed a significant PFS benefit that was consistent across subgroups with PD-L1 <1, 1 - 49% and ≥50% (HRs = 0.47, 0.56 and 0.45, respectively [all P<0.003]). Although the OS data from CHOICE-01 were immature, they also demonstrated benefit that was consistent across subgroups with PD-L1 <1, 1 - 49% and ≥50% (HRs = 0.79, 0.72 and 0.82, respectively [all P>0.05]).

## 4 Discussion

Preclinical data indicate that toripalimab activity is potentially independent of PD-L1 expression. The enhanced binding and likely consequential greater T-cell activation of toripalimab provide evidence to support this hypothesis. The phase 3 RCT data described in this manuscript demonstrate that: 1] addition of a PD-1 inhibitor to conventional chemotherapy provides survival benefit in untreated, advanced NPC, ESCC, and NSCLC; and 2] some PD-1 inhibitors assessed provided benefit among patients with low/no PD-L1 expression in NPC and NSCLC albeit to a lesser degree than those with high/intermediate PD-L1 expression. These findings are consistent with prior reports (28) and were not the focus of the current analysis which was to assess whether any of the PD-1 inhibitors combined with chemotherapy demonstrated potential differentiating features such as the ability to provide survival benefit independent of PD-L1 expression based on available phase 3 RCT data. Across studies in untreated, advanced ESCC, we observed that the survival benefits of adding toripalimab to chemotherapy in JUPITER-06 were independent of PD-L1 expression level, results that were unique among the individual phase 3 studies in advanced ESCC. Similarly, the OS benefit with toripalimab for advanced ESCC with PD-L1 <1 reported in JUPITER-06 distinguishes it from a pooled analysis of patient-level data from the phase 3 RCTs for nivolumab, pembrolizumab and tislelizumab performed by the US FDA which found no clear OS benefit from treatment with these PD-1 inhibitors (18). Findings of similar or greater benefit among patients with low/no PD-L1 expression versus high/intermediate PD-L1 expression with toripalimab plus chemotherapy were also observed in advanced NPC (JUPITER-02) and NSCLC (CHOICE-01) for both OS and PFS. Whether toripalimab truly provides unique clinical benefit independent of PD-L1 expression compared with other PD-1 inhibitors remains currently unknown due to the limitations associated with indirect comparison of RCTs.

As previously discussed, we focused our analyses on phase 3 RCT data because of the relatively comparable study designs/populations, large sample sizes and availability of survival data across studies. A shortcoming of this approach was the absence of phase 3 head-to-head data which meant comparisons of the different PD-1 inhibitors was strictly indirect in nature. However, our literature search did identify one randomized head-to-head phase 2 trial (PERLA) which investigated either dostarlimab or pembrolizumab added to chemotherapy for untreated, metastatic NSQ NSCLC (7). Although PERLA was not powered to detect statistical differences, numerical differences in benefit across numerous endpoints favoring the dostarlimab versus pembrolizumab arm were reported (objective response rate [primary endpoint], OS, PFS, PFS across all categories of PD-L1 expression) suggesting potential differentiation between these PD-1 inhibitors.

The major limitation of our interpretations is that they are based on indirect comparisons across different clinical trials. The trials included in our analyses varied with regard to the: 1] patient populations (some studies only included patients from Asia [predominantly China] while others included global populations); 2] determination of PD-L1 expression (a variety of different assays and cell populations were utilized with some studies counting only tumor cells while others counted both tumor and immune cells); 3] treatment regimens (the specific chemotherapeutic agents, number of cycles administered, and inclusion/exclusion of chemotherapy during maintenance treatment was variable across trials); 4] duration of follow-up (only interim analyses with relatively limited follow-up were available for some studies which may have impacted the magnitude of survival benefit reported). These limitations prevent definitive identification of superiority for one mAb vs the others, but could serve as support for clinical decision-making. Our results are therefore suggestive but not conclusive with head-to-head clinical trials comparing PD-1 inhibitors required for more definitive conclusions to be drawn.

## 5 Conclusions

In conclusion, preclinical data and indirect comparison of results across phase 3 RCTs in advanced NPC, ESCC, and NSCLC suggest that toripalimab differentiates itself from other PD-1 inhibitors by providing survival benefit that is relatively independent of tumor PD-L1 expression. However, determination of clinically-meaningful differences requires sufficiently powered head-to-head RCTs, which would require substantial resources and time but potentially optimize therapy selection among patients with low/no PD-L1 expression. Additional areas of further study with PD-1 inhibitor therapy among patients with advanced cancer to further improve outcomes also include those seeking to elucidate genomic information/biomarkers that may identify more effective novel treatment approaches including PD-1 inhibitor-based combinations, as well as the optimal sequence and duration of treatment.
